# Network Pharmacology and Experimental Validation Reveal the Effects of Chidamide Combined With Aspirin on Acute Myeloid Leukemia-Myelodysplastic Syndrome Cells Through PI3K/AKT Pathway

**DOI:** 10.3389/fcell.2021.685954

**Published:** 2021-09-09

**Authors:** Simin Liang, Xiaojia Zhou, Duo Cai, Fernando Rodrigues-Lima, Jianxiang Chi, Li Wang

**Affiliations:** ^1^Department of Hematology, The First Affiliated Hospital of Chongqing Medical University, Chongqing, China; ^2^Department of Hematology, The Third Affiliated Hospital of Chongqing Medical University, Chongqing, China; ^3^Université de Paris, Unité de Biologie Fonctionnelle et Adaptative, CNRS UMR 8251, Paris, France; ^4^Center for the Study of Hematological Malignancies, Karaiskakio Foundation, Nicosia, Cyprus

**Keywords:** chidamide, aspirin, network pharmacology, myelodysplastic syndromes, PI3K/Akt pathway

## Abstract

Chidamide (CDM), a novel histone deacetylase inhibitor, is currently used for patients with peripheral T-cell lymphoma. Aspirin (ASA), an anti-inflammatory drug, has been shown to exert anticancer activity. Herein, we investigated the effect of CDM combined with ASA on myelodysplastic syndromes-derived acute myeloid leukemia (AML-MDS) cells and explored the underlying mechanism. The putative targets of CDM and ASA were predicted by network pharmacology approach. GO functional and KEGG pathway enrichment analyses were performed by DAVID. Furthermore, experimental validation was conducted by Cell Counting Kit-8 assay, Flow cytometry and Western blotting. Network pharmacology analysis revealed 36 AML-MDS-related overlapping genes that were targets of CDM and ASA, while 10 hub genes were identified by the plug-in cytoHubba in Cytoscape. Pathway enrichment analysis indicated CDM and ASA significantly affected PI3K/AKT signaling pathway. Functional experiments demonstrated that the combination of CDM and ASA had a remarkable synergistic anti-proliferative effect by blocking the cell cycle in G0/G1 phase and inducing apoptosis. Mechanistically, the combination treatment significantly down-regulated the phosphorylation levels of PI3K and AKT. In addition, insulin-like growth factor 1 (IGF-1), an activator of PI3K/AKT pathway, reversed the effects of the combination treatment. Our findings suggested that CDM combined with ASA exerted a synergetic inhibitory effect on cell growth by inactivating PI3K/AKT pathway, which might pave the way for effective treatments of AML-MDS.

## Introduction

Myelodysplastic syndromes (MDS) are a heterogeneous group of myeloid disorders characterized by ineffective hematopoiesis, peripheral blood cytopenia and high risk of transformation to acute myeloid leukemia (AML) with poor prognosis (Cogle et al., 2011; Scalzulli et al., 2020). MDS-derived AML (AML-MDS) shows slower hematologic recovery and poorer outcomes following intensive remission-induction chemotherapy than *de novo* AML (Boddu et al., 2017; Ramadan et al., 2020). Additionally, allogeneic stem cell transplantation (alloSCT) remains the only curative option for patients with AML-MDS. But unfortunately, it is only suitable for a minority (Schroeder et al., 2019). Therefore, a novel effective treatment strategy with minimal cytotoxicity still needs to be developed for AML-MDS.

Chidamide (CDM), a novel histone deacetylase inhibitor, selectively inhibits HDAC1, 2, 3, and 10, and has been approved for treatment of patients with recurrent or refractory peripheral T cell lymphoma (PTCL) in China (Shi et al., 2015; Lu et al., 2016). Strikingly, a number of studies have suggested that CDM exerts cytotoxic effects on lymphoma (Zhou et al., 2018), multiple myeloma (MM) (Sun et al., 2019), MDS (Liu et al., 2016), and leukemia (Li et al., 2015), as well as non-hematological malignancies, including lung cancer (Wu et al., 2019), colon cancer (Liu et al., 2010) and hepatocellular carcinoma (Wang et al., 2012). Moreover, CDM has been shown to synergize effects with other anti-tumor agents. For example, several studies have showed that CDM combined with hypomethylating agents, including decitabine, resulted in synergistic effects on the proliferation and apoptosis of myeloid leukemia cells (Xu et al., 2019; Li et al., 2020). Co-treatment with CDM and Bortezomib reduced proliferation, invasion and migration of gastric cancer cells (Zhang et al., 2020). Co-treatment with CDM and Rituximab inhibited tumor growth by upregulating CD20 in diffuse large B-cell lymphoma (DLBCL) (Guan et al., 2020).

Aspirin (acetylsalicylic acid, ASA) has been widely used as an anti-inflammatory, analgesic drug, as well as in cardiovascular disease and platelet aggregation. ASA can reduce the morbidity and mortality of several malignancies, including gastric cancer (García Rodríguez et al., 2020), lung cancer (Erickson et al., 2018) and prostate cancer (Hurwitz et al., 2019). Recent studies revealed that ASA combined with other drugs, such as sorafenib and atorvastatin, exhibited strong anti-cancer effects *in vitro* and *in vivo* (Pennarun et al., 2013; He et al., 2017). In addition, since ASA could affect histone methylation, we aimed to investigate the potential effects and mechanisms of CDM combined with ASA on AML-MDS.

**FIGURE 1:**
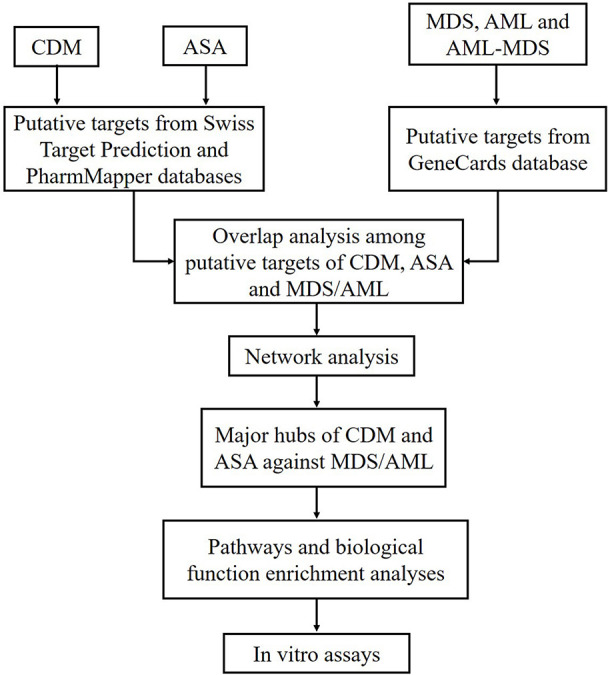
Flowchart of the study design based on network pharmacology approaches for deciphering the mechanisms of CDM and ASA acting on AML-MDS. CDM, Chidamide; ASA, Aspirin; MDS, myelodysplastic syndrome; AML, acute myeloid leukemia; AML-MDS, myelodysplastic syndromes-derived acute myeloid leukemia.

Recently, network pharmacology has been used to predict the therapeutic targets and efficacy of drugs by constructing drug-drug, drug-target and other networks, using a variety of database resources. Through preliminary experiment, we found that CDM and ASA had synergistic inhibitory effect on cell growth in leukemia cells. In this study, we aimed to investigate the anti-tumor activity of CDM combined with ASA in AML-MDS, explore underlying mechanisms by predicting related targets through the network pharmacology approach, so as to provide theoretical and experimental basis for the treatment of AML-MDS. The flowchart of this study design was presented in Figure 1.

## Materials and Methods

### Target Prediction Based on Network Pharmacology

SwissTargetPrediction^1^ and PharmMapper^2^ were used to establish the targets of CDM and ASA. Genomic targets of MDS, AML and AML-MDS were obtained from GeneCards^3^ and overlapping genes were collected. Subsequently, CDM- and ASA-associated targets were mapped to these overlapping disease-targets, followed by therapeutic targets of CDM and ASA against AML-MDS were obtained. The STRING database^4^ was used to obtain interactions among potential targets of CDM, ASA and the aforementioned diseases. Protein interactions with a combined score > 0.4 were selected. Cytoscape 3.2.1 was used to construct and analyze the protein-protein interaction (PPI) network. DAVID database^5^ was used to perform Gene Ontology (GO) and Kyoto Encyclopedia of Genes and Genomes (KEGG) pathway enrichment analyses.

### Reagents and Antibodies

CDM (CS055, purity > 95%) was supplied by Chipscreen Biosciences (Shenzhen, China), while ASA was obtained from Maclin (A800349, Shanghai, China). IGF-1 was purchased from MedChemExpress (MCE, Shanghai, China). The following primary antibodies were used: rabbit anti-Bcl-2 (YT0470), cleaved Caspase-3 (YC0006) from ImmunoWay (Texas, United States). PI3K (bsm-33219M), p-PI3K (AB1235888), and Caspase-3 (bs-0081R) from Bioss (Beijing, China). AKT (4691T), p-AKT (4060T), p21^CIP1^ (2947T) from Cell Signaling Technology (Danvers, United States). CDK2 (H08211543) and CDK4 (H10082274) from Wanleibio (Shenyang, China). Mouse anti-β-actin (KM9001) from Sungene (Tianjin, China). Horseradish peroxidase (HRP)-conjugated goat anti-rabbit IgG (A0239) and anti-mouse IgG (A0258) were purchased from Beyotime Biotechnology (Shanghai, China).

### Cell Lines and Cell Culture

The human AML-MDS cell line, SKM-1, was a gift from Professor Jianfeng Zhou working in Tongji Medical College of Huazhong University of Science and Technology (Wuhan, China), while the T cell acute lymphoblastic leukemia (T-ALL) cell line Molt-4 was provided by the Children’s Hospital of Chongqing Medical University (Chongqing, China). The cells were maintained in RPMI-1640 (Gibco, Thermo Fisher Scientific, MA, United States) supplemented with 10% fetal bovine serum (PAN seratech, Germany) and 100 U/ml penicillin and 100 μg/ml streptomycin (1 × P/S).

### Cell Viability Assay

Cells in the logarithmic growth phase were seeded at a density of 1,500 cells/well and cultured overnight. Drugs were administered at 0.1 μL per well. After a 72-h incubation, cell viability was measured using Cell Titer-Glo luminescent cell viability assay kit (Promega, Madison, United States) and luminescence was quantified using Envision Plate-Reader.

Cell viability was also measured by Cell Counting kit-8 (CCK-8) assay (MCE, Shanghai, China). Briefly, cells were seeded at 3,000 cells/100 μL and treated with different concentrations of CDM and ASA for 24, 48, and 72 h. CCK-8 reagent was added and incubated for 3 h. The absorbance at 450 nm was measured using a Multiskan Go Microplate Spectrophotometer (Thermo Fisher Scientific, United States). Cell proliferation inhibition rate was calculated based on the formula: absorbance of (control group − experimental group)/absorbance of (control group – blank group) × 100%.

### Cell Cycle and Cell Apoptosis

CDM, at a concentration of 0.5 μM, or ASA, at a concentration of 1 mM, was added to 1 million cells for 48 h. For cell cycle analysis, cells were fixed with ice-cold 75% ethanol overnight at 4°C and then incubated with 50 mg/ml of propidium iodide (PI) for 30 min at room temperature. For apoptosis, cells were incubated with 5 μl of Annexin V-FITC and 10 μl of PI, at 4°C for 15 min in the dark. Cell cycle and apoptosis were analyzed using a flow cytometer (CytoFLEX, Beckman Coulter, United States).

### RNA Isolation and Reverse Transcription-Quantitative PCR (RT-qPCR)

Total RNA was extracted from cells using TRIzol reagent (Beyotime, China) according to the manufacturer’s instructions. cDNA was synthesized using PrimeScript Reverse Transcription reagent kit (Takara, Japan). Quantitative PCR (qPCR) was performed using a CFX96 Touch^TM^ Real-Time PCR Detection System (Bio-Rad, Hercules, CA, United States). The following RT-qPCR parameters were used: 95°C for 30 s; 95°C for 5 s, and 60°C for 30 s repeated over 40 cycles. All primers were synthesized by Tsingke (Beijing, China) and the sequences were as follows: P21 forward: 5′-CTGCCTTAGTCTCAGTTTGTGT-3′; P21 reverse: 5′-AACCTCTCATTCAACCGCCTA-3′; Bcl-2 forward: 5′-CTGCACCTGACGCCCTTC-3′; Bcl-2 reverse: 5′- ACACATGACCCCACCGAAC-3′; caspase-3 forward: 5′-TGC TGAAACAGTATGCCGACA-3′; caspase-3 reverse: 5′-CAAAT TCTGTTGCCACCTTTCG-3′; β-actin forward: 5′-CCCAAA GTTCACAATGTGGC-3′; β-actin reverse: 5′-GACTTCCTGT AACAACGCATC-3′. Transcript levels were normalized to β-actin expression and the target gene expression was calculated using the formula 2^–ΔΔCt^.

### Western Blot Analysis

Total protein from the cells was harvested using RIPA lysis buffer supplemented with 1 μM PMSF (Beyotime, Shanghai, China) and 30 μg protein was separated on a 10% SDS-polyacrylamide gradient gel. The proteins were transferred onto PVDF membranes and blocked with 5% non-fat milk in Tris Buffered Saline with Tween-20 (TBST) for 2 h at room temperature. The blots were then incubated with primary antibodies overnight at 4°C. Membranes were then washed 3 times with TBST and incubated with secondary antibodies for 1 h at room temperature. Protein bands were visualized with an ECL kit (Advansta, United States) and the band intensity was analyzed using Vilber Fusion software (Fusion, FX5 Spectra, France). β-actin was used as a loading control.

### Statistical Analysis

All data was presented as means ± standard deviation (SD) and statistical analyses were performed using GraphPad Prism 5.01 (GraphPad Software Inc., San Diego, CA, United States). The results were analyzed using one-way and two-way ANOVA followed by the Bonferroni *post hoc* test. A value of *p* < 0.05 was considered as statistically significant. All experiments were performed in triplicates.

## Results

### Putative Targets of CDM and ASA for the Treatment of AML-MDS

**FIGURE 2:**
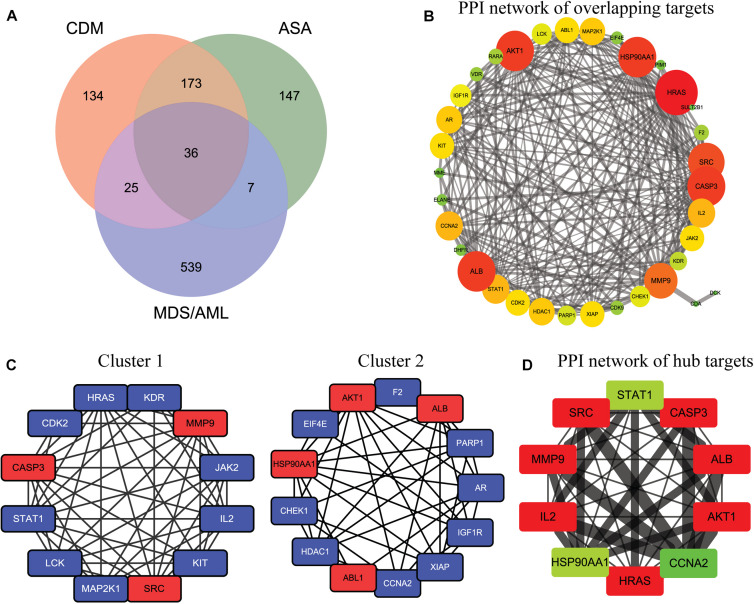
The network pharmacology of CDM and ASA against AML-MDS and cluster analysis. **(A)** The Venn diagram of the potential targets of CDM and ASA against AML-MDS. **(B)** A PPI network of the overlapping targets of CDM and ASA against AML-MDS by Cytoscape software. **(C)** Clusters of interacted proteins by use of MCODE algorithm. **(D)** Hub targets of the PPI network by use of cytoHubba. The size of node and edge was mapped to the degree and edge betweenness, respectively. The color of the node represents the size of the degree value. The redder the color, the larger the node and the more important it is in the network. Conversely, the greener the color, the smaller the node and the less important it is in the network.

A total of 522 possible targets of CDM and ASA were predicted by Swiss Target Prediction and PharmMapper (Supplementary Table 1), and 607 overlapping targets of MDS, AML and AML-MDS were obtained from the GeneCards database (Supplementary Table 2). Ultimately, 36 targets of CDM and ASA against AML-MDS were collected (Figure 2A and Supplementary Table 3). A PPI network of these predicted targets was analyzed using STRING database and constructed by Cytoscape, and the network contained 35 nodes and 245 edges (Figure 2B). Clustering subnetworks were produced using the MCODE algorithm (Figure 2C). Specifically, 10 nodes were identified as hub genes by the cytoHubba plugin in Cytoscape and grouped together, including AKT1, ALB, CASP3, SRC, MMP9, IL2, HRAS, CCNA2, STAT1, HSP90AA1 (Figure 2D and Supplementary Table 4).

### Biological Function and Pathway Enrichment Analyses of Hub Targets

**FIGURE 3:**
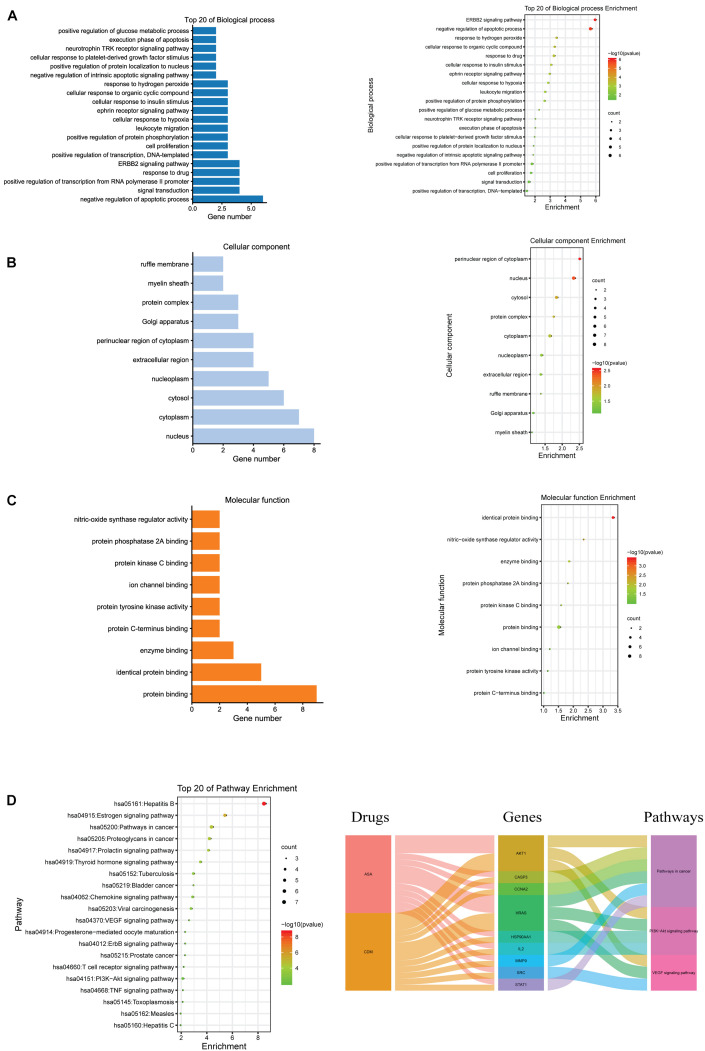
GO and KEGG pathway enrichment analyses for the hub genes of CDM and ASA against AML-MDS. **(A)** Biological process (BP); **(B)** cellular component (CC); **(C)** molecular function (MF); **(D)** the top 20 of KEGG enrichment analysis (left panel), alluvial plot of interaction among drugs, major hub genes and three main pathways (right panel).

GO and KEGG pathway enrichment analyses of these hub genes were performed using the DAVID database. GO analysis showed that these targets were associated with negative regulation of apoptotic processes and cell proliferation (Figure 3A). Enrichment in cellular component and molecular function was presented in Figures 3B,C, respectively. Additionally, KEGG analysis revealed that these 10 hub targets were involved in 42 pathways, which were mainly enriched in cancer-related pathways, especially PI3K/AKT and VEGF signaling pathways (Supplementary Table 5 and Figure 3D).

### The Synergistic Antiproliferative Effects of CDM Combined With ASA on AML-MDS

**FIGURE 4:**
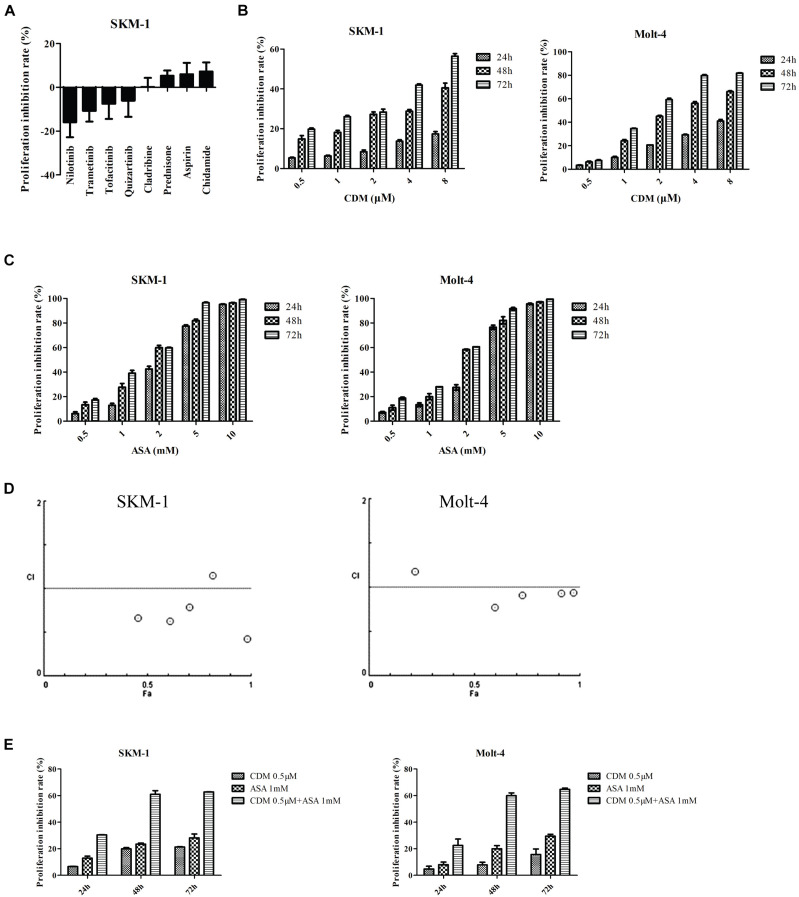
CDM in combination with ASA inhibited the proliferation of AML-MDS cells. **(A)** Cell viability detected by Cell Titer-Glo luminescent cell viability assay in SKM-1 cells treated with 8 drugs. **(B,C)** SKM-1 and Molt-4 cells were exposed to CDM (0.5, 1, 2, 4, and 8 μM) or ASA (0.5, 1, 2, 5, and 10 mM) alone. **(D)** Combination index values were calculated with CompuSyn software. CI < 1 indicates synergy; CI = 1 is additive; and CI > 1 means antagonism. CI, combination index; Fa, effect levels. **(E)** SKM-1 and Molt-4 cells were treated with 0.5 μM CDM combined with 1 mM ASA for 24, 48, and 72 h. The cell viability was determined by CCK-8 assay. Data are mean ± SD of three independent experiments.

Then sensitivity of SKM-1 cells to the drugs was determined according to the converting plasma concentrations. The results showed that SKM-1 cells were sensitive to CDM and ASA (Figure 4A). For further validation, SKM-1 and Molt-4 cells were treated with different concentrations of the two drugs alone. The results of CCK-8 assay showed that both CDM and ASA inhibited cells viability in a dose- and time-dependent manner (Figures 4B,C). At 48 h, the half-maximal inhibitory concentration (IC_50_) of CDM on SKM-1 and Molt-4 cells was (19.54 ± 3.34) μM and (1.69 ± 0.08) μM, respectively, and IC_50_ value of ASA was (1.69 ± 0.06) mM and (1.84 ± 0.08) mM, respectively. Moreover, to evaluate the synergistic effect of co-treatment on cell viability, these cells were treated with a low-dose CDM (0.5 μM) combined with different concentrations of ASA for 48 h. When combined with ASA, low-dose CDM decreased the IC_50_ of ASA to (0.63 ± 0.06) mM and (0.94 ± 0.05) mM in SKM-1 and Molt-4 cells, respectively. The combination index (CI) demonstrated that CDM combined with ASA had a distinct synergistic effect calculated by CompuSyn software (Figure 4D). As shown in Figure 4E, CDM combined with ASA significantly enhanced the inhibitory effect. Therefore, a combination of 0.5 μM CDM and 1 mM ASA, a value close to the IC_50_, was selected for subsequent experiments.

### Combination of CDM and ASA Caused Cell Cycle Arrest at the G0/G1 Phase

**FIGURE 5:**
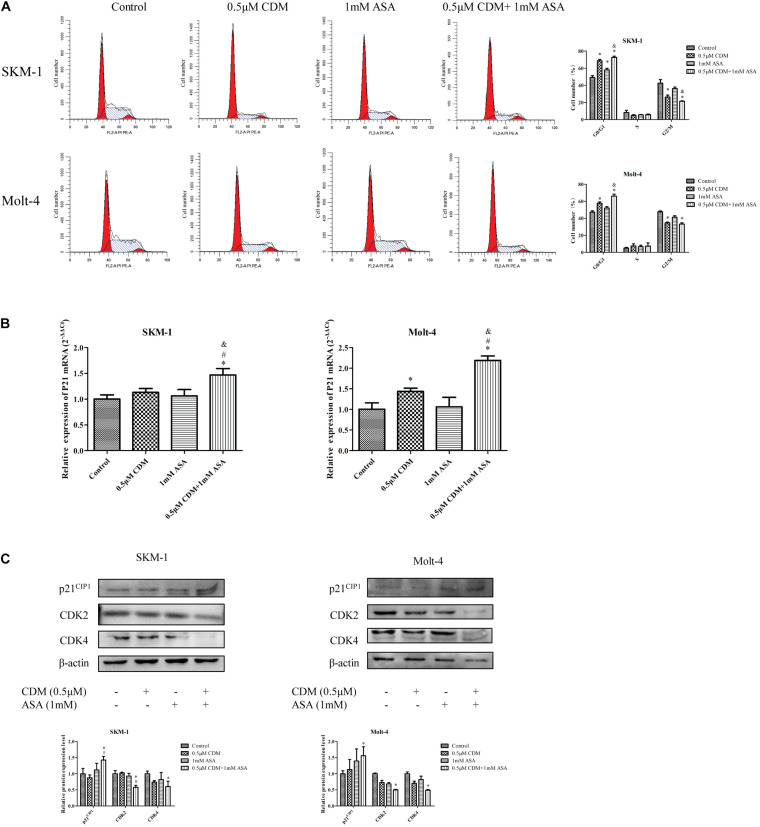
CDM in combination with ASA arrested the cell cycle at G0/G1 phase in AML-MDS cells. **(A)** Cell cycle distribution was detected by flow cytometry after treatment with 0.5 μM CDM and 1 mM ASA for 48 h. The red color areas on the left and right of the images represent the proportion of cells in the G0/G1 and G2/M phases, respectively. **(B)** Relative expression of P21 mRNA compared with the control group. **(C)** Expressions of cell cycle related protein (CDK2, CDK4, p21) were detected by western blot. β-actin was used as a loading control. Data are mean ± SD of three independent experiments. “^∗^” indicates a significant difference relative to the control group (^∗^*p* < 0.05), “#” indicates a significant difference relative to CDM-treated group (^#^*p* < 0.05), “&” indicates a significant difference relative to ASA-treated group (^&^*p* < 0.05).

To investigate the efficacy of the combination treatment on cell cycle, cell cycle distribution of each group was detected by flow cytometry with PI staining assay. As shown in Figure 5A, the combination of CDM and ASA resulted in a significant increase in the proportion of G0/G1 phase cells compared with the two drugs alone. A high mRNA expression of p21 in the combined treatment group (Figure 5B) was observed, but there was no statistical difference between the two mono-treatment groups. Also, Western blotting indicated that after combined treatment, the protein expression of CDK2 and CDK4 was down-regulated while p21 was up-regulated (Figure 5C).

### Combination of CDM and ASA Synergistically Induced Apoptosis

**FIGURE 6:**
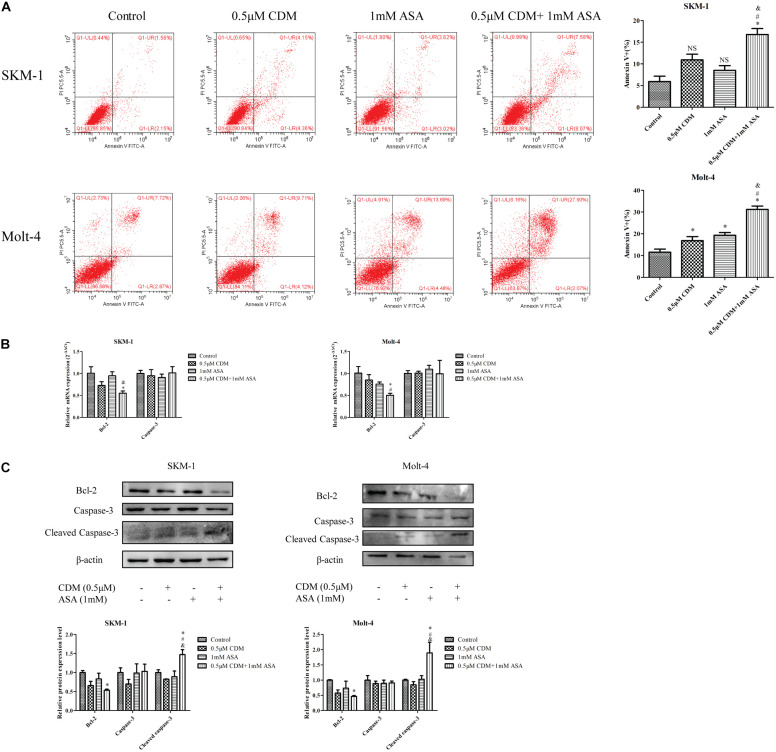
CDM in combination with ASA induced cell apoptosis in AML-MDS cells. **(A)** Cell apoptotic rate was detected by flow cytometry after treatment of the combination of 0.5 μM CDM and 1 mM ASA for 48 h. **(B)** Relative mRNA expression of Bcl-2 and caspase-3 compared with the control group. **(C)** Expressions of the apoptosis-related protein (Bcl-2, caspase-3, cleaved caspase-3) were detected by western blot. β-actin was used as a loading control. Data are mean ± SD of three independent experiments. “^∗^” indicates a significant difference relative to the control group (^∗^*p* < 0.05), “#” indicates a significant difference relative to CDM-treated group (^#^*p* < 0.05), “&” indicates a significant difference relative to ASA-treated group (^&^*p* < 0.05), “NS” indicates no significant difference relative to ASA-treated group or CDM-treated group.

Next, flow cytometry was used to detect the apoptotic effects of the combination treatment on SKM-1 and Molt-4 cells, and apoptosis rate was calculated as the sum of percentage of Annexin^+^ cells. As shown in Figure 6A, the co-treatment remarkably induced apoptosis compared with CDM and ASA mono-treatment. Furthermore, Western blotting showed that the expression of apoptosis-related protein cleaved caspase-3 was up-regulated and the expression of Bcl-2 was down-regulated, while no change in caspase-3 was observed. The observation was consistent with the results of RT-qPCR (Figures 6B,C).

### Combination of CDM and ASA Suppressed PI3K/AKT Signaling Pathway

**FIGURE 7:**
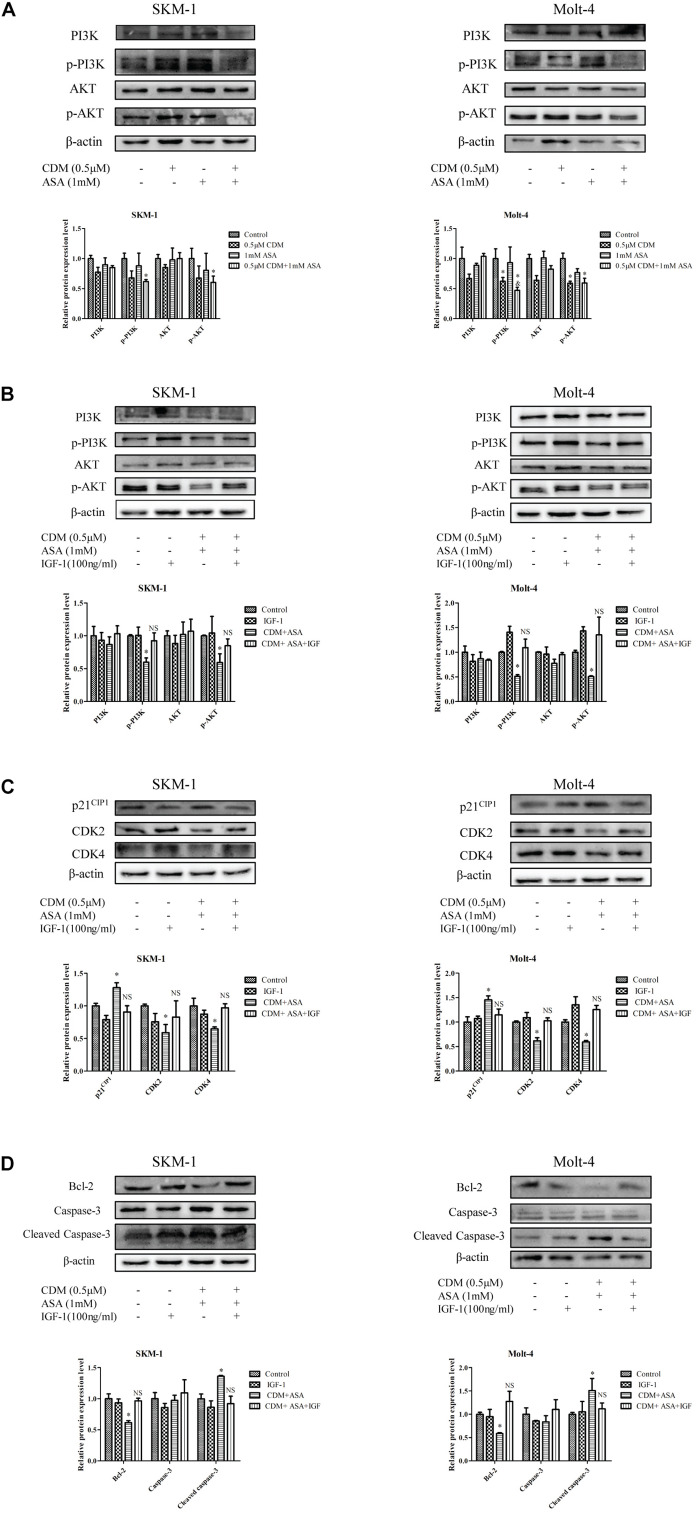
CDM combined with ASA inhibited the activation of PI3K/AKT pathway in AML-MDS cells. **(A)** Western blot analysis of PI3K, p-PI3K AKT, p-AKT. **(B)** Western blotting of PI3K, p-PI3K, AKT and p-AKT. **(C)** Western blotting of CDK2, CDK4 and p21. **(D)** Western blotting of Bcl-2, caspase-3 and cleaved caspase-3. IGF-1 reversed the effect of CDM combined with ASA on AML-MDS cells. β-actin served as a loading control. Data are mean ± SD of three independent experiments. “^∗^” indicates a significant difference relative to the control group (^∗^*p* < 0.05), “&” indicates a significant difference relative to ASA-treated group (&*p* < 0.05), “NS” indicates no significant difference relative to the control group.

Based on the results of GO and KEGG analyses, we focused on PI3K/AKT signaling pathway and hypothesized that the combination of CDM and ASA could synergistically inhibit the activation of PI3K/AKT pathway. The results of Western blotting demonstrated that the expression levels of p-PI3K and p-AKT in the combination treatment group were distinctly lower than those in each drug alone and control group, while the level of total PI3K and AKT remained constant (Figure 7A). Intriguingly, IGF-1, an agonist of PI3K/AKT signaling pathway, reversed the effects of the combination treatment on cell cycle and apoptosis-related proteins (Figures 7B–D), indicating that PI3K/AKT pathway was involved in the process induced by the combination treatment of CDM and ASA.

## Discussion

Drug combination has been proposed as a promising therapeutic strategy with fewer side effects and lower toxicity, which could be used to improve the efficacy of single-agent treatment. There was evidence that ASA synergized with HDAC inhibitors (FK228) to inhibit growth of COX-1 positive ovarian cancer cells (Son et al., 2010). Zhang H. et al. (2018) also reported that in the presence of CDM, ASA significantly suppressed tumor growth of natural killer/T-cell lymphoma (NKTCL). In line with previous reports, our study showed that the efficiency of co-treatment was superior to CDM or ASA mono-treatment alone in inhibiting growth of AML-MDS cells, suggesting that a small dose of drug combination may be an effective therapy. However, the underlying molecular mechanisms remain unclear.

To identify the mechanisms in which CDM and ASA affected cell viability, we examined the cell cycle distribution and apoptotic rate. Dysregulation of cell cycle progression is a hallmark of cancer that enables limitless cell division. It has been reported that CDM and ASA inhibit tumor cell proliferation by inducing cell cycle arrest in G0/G1 and G2/M phases (Liu et al., 2016; Fan et al., 2017; He et al., 2018; Zhang X. et al., 2018). Our data showed that the efficiency of inducing cell cycle arrest was significantly improved when CDM was combined with ASA. The interactions of cyclin, cyclin-dependent kinases (CDKs) and CDK inhibitors play indispensable roles in controlling cell cycle. CDK2 is necessary for transition from G1 phase to S phase, while CDK4 controls G1 phase, both of which are positive regulators of the cell cycle (Lim and Kaldis, 2013). On the contrary, p21, a putative tumor-suppressor protein, is negative regulator that inhibits the CDKs/cyclin complexes in the G1 phase (Rodriguez-Cupello et al., 2020). Our study manifested that the combination of CDM and ASA synergistically down-regulated CDK2 and CDK4, and up-regulated p21, leading to G0/G1 arrest.

Dysregulation of apoptosis causes excessive cell proliferation or excessive apoptosis, resulting in disease. As a signaling pathway that regulates cell apoptosis and survival, the Bcl-2/Cleaved caspase-3 apoptotic pathway has been implicated in many cancers including leukemia (Tian et al., 2019; Zhang et al., 2019). Bcl-2 is a member of anti-apoptotic Bcl-2 family proteins, which plays an important role in maintaining the integrity of the outer mitochondrial membrane (OMM), while the pro-apoptotic protein Bax inserts into the OMM and facilitates the release of inter-membrane space (IMS) protein, leading to the activation of caspases (Renault and Chipuk, 2014; Delbridge et al., 2016). Cleaved caspase-3 is an activated form of caspase-3, a major effector protease in apoptosis that triggers the apoptotic cascade (Braunstein et al., 2020). We found that CDM combined with ASA significantly accelerated cell apoptosis by downregulation of Bcl-2 and activation of caspase-3, indicating that the combination treatment might be a potential strategy for the treatment of leukemia.

**FIGURE 8:**
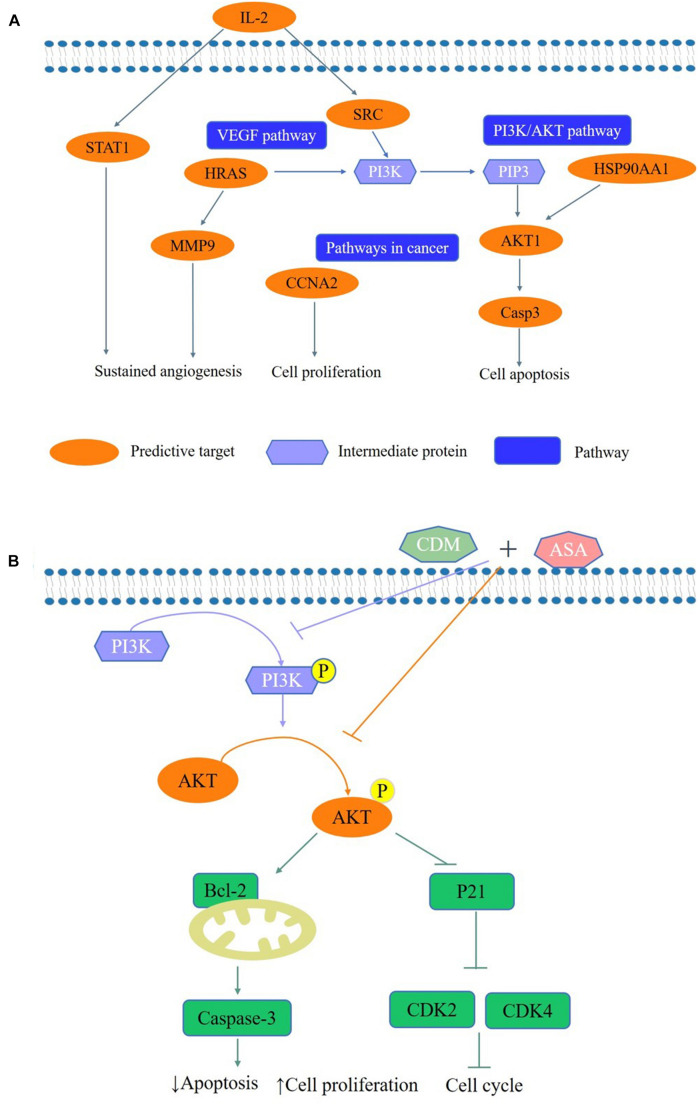
A schematic representation of the proposed pathway responsible for CDM combined with ASA in AML-MDS cells. **(A)** The predictive pathways of CDM and ASA in AML-MDS through network pharmacology. **(B)** CDM combined with ASA could inhibited the activation of PI3K/AKT signaling pathways, and then affected the expression of cell cycle and apoptosis-related proteins to induce cell cycle arrest and apoptosis in AML-MDS cells through experimental validation.

Based on the network pharmacology approach, we collected 36 putative targets of CDM and ASA against AML-MDS, and revealed that AKT1 was one of the hub genes. Through KEGG enrichment analysis, PI3K/AKT signaling pathway was highlighted as a potential target. The expression of PI3K/AKT signaling pathway is often dysregulated in various cancers and activated PI3K/AKT pathway is implicated in a variety of processes, including inducing tumor cell proliferation, inhibiting apoptosis and promoting invasion and metastasis (Yang et al., 2019). Previous studies have confirmed that blocking PI3K/AKT pathway induces cell death by regulating cell proliferation, apoptosis and cell cycle in leukemia (Bertacchini et al., 2015; Banerjee et al., 2016; Cheng et al., 2019). CDM was able to increase the acetylation levels of histone H3 and inhibit PI3K/AKT signaling pathway, resulting in arresting colon cancer cells at G1 phase and accelerating cell apoptosis (Liu et al., 2010). ASA was shown to inhibit cell proliferation by blocking cell cycle by suppressing the activation of the phosphorylation of AKT (Zhang X. et al., 2018). Consistently, our data showed that the expression levels of p-PI3K and p-AKT were remarkably downregulated by the combination of CDM and ASA, leading to the inactivation of the PI3K/AKT pathway. To confirm this, cells were treated with IGF-1, a PI3K/AKT agonist. The results showed that IGF-1 reversed the inhibitory effect of the combination treatment on PI3K/AKT pathway. The above results suggested that the combination of CDM and ASA inhibited cell proliferation, induced cell cycle arrest and promoted apoptosis in AML-MDS cells partially through suppressing the PI3K/AKT pathway (Figure 8).

## Conclusion

Our study demonstrated that CDM and ASA exerted synergistic effect on G0/G1 arrest and apoptosis by inhibiting the PI3K/AKT pathway *in vitro*. This provides a promising chemotherapeutic strategy for AML-MDS in combination with low dose agents. Future studies should focus on the *in vivo* efficacy of the combination treatment and the determination of the optimal combination regimens.

## Data Availability Statement

The original contributions presented in the study are included in the article/Supplementary Material, further inquiries can be directed to the corresponding author/s.

## Author Contributions

SL and XZ conceived and designed the experiments. SL, XZ, and DC performed the experiments. LW contributed as regards the reagents, materials, and analysis tools. SL, XZ, DC, FR-L, JC, and LW participated to the analysis and interpretation of the results. SL wrote the manuscript. All authors reviewed and approved the final manuscript.

## Conflict of Interest

The authors declare that the research was conducted in the absence of any commercial or financial relationships that could be construed as a potential conflict of interest.

## Publisher’s Note

All claims expressed in this article are solely those of the authors and do not necessarily represent those of their affiliated organizations, or those of the publisher, the editors and the reviewers. Any product that may be evaluated in this article, or claim that may be made by its manufacturer, is not guaranteed or endorsed by the publisher.

## Footnotes

1
http://swisstargetprediction.ch/

2
http://www.lilab-ecust.cn/pharmmapper/index.html

3
https://www.genecards.org/

4
http://string-db.org/

5
http://David.ncifcrf.gov/
